# Evaluating the eighth edition TNM staging system for esophageal cancer among patients receiving neoadjuvant therapy: A SEER study

**DOI:** 10.1002/cam4.2997

**Published:** 2020-05-11

**Authors:** Yonggang Yuan, Ge Ma, Xuelei Hu, Qingyuan Huang

**Affiliations:** ^1^ Department of Thoracic Surgery Qilu Hospital of Shandong University(Qingdao) Qingdao P.R. China; ^2^ Department of Respiratory Medicine Yidu Central Hospital of Weifang Weifang China; ^3^ Shanghai First People's Hospital Shanghai Jiao Tong University Shanghai China

**Keywords:** esophageal cancer, neoadjuvant therapy, tumor grade, ypTNM staging

## Abstract

**Background:**

The evaluation of the eighth edition of ypTNM staging system for patients with esophageal cancer was limited in the setting of neoadjuvant therapy.

**Methods:**

A total of 2324 patients with esophageal cancer receiving radio(chemo)therapy prior to surgery from the Surveillance, Epidemiology, and End Results (SEER) database between 2004 and 2013 were eligible for the analysis. Kaplan‐Meier method and Cox proportional hazards models were used to estimate overall survivals.

**Results:**

Among patients with preoperative therapy, both the seventh edition TNM grouping and the eighth edition ypTNM grouping could significantly stratify the overall survival (both log‐rank *P* < .001). There was not significant difference in the *C*‐index of the seventh edition TNM grouping (0.575; 95%CI, 0.558‐0.593) and the eighth edition ypTNM grouping (0.569; 95%CI, 0.551‐0.587) (*P* = .098). In multivariable Cox analysis, ypN category was the strongest predictor of overall survival (*P* < .001), followed by tumor grade (HR, 1.33; 95%CI, 1.12‐1.56; *P* = .001). The combination of ypT, ypN, and ypG categories yielded significantly higher *C*‐index (0.591; 95%CI, 0.573‐0.609) than that of the seventh edition TNM staging (*P* = .024).

**Conclusion:**

Tumor grade remained an independent predictor of overall survival in the setting of neoadjuvant therapy, and could improve the performance of ypTNM staging system.

## Graphical abstract

Analysis of the national SEER database revealed that lymph node status (ypN category) was an important prognostic factor, and supported the combination of ypT1 and ypT2 in grouping. Tumor grade ramained an independent predictor of survival in the setting of neoadjuvant therapy, and could improve the performance of ypTNM staging system.

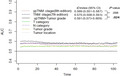

## 1 INTRODUCTION

During the past decades, esophageal adenocarcinoma (EAC) has experienced a rapid increase of incidence in North America and Europe, while esophageal squamous‐cell carcinoma (ESCC) remains the predominant subtype in Asia, Africa, and South America.^1^ Prospective randomized clinical trials and meta‐analyses have demonstrated neoadjuvant radiochemotherapy could substantially prolong the survival of patients with locally advanced disease.^2^, ^3^, ^4^, ^5^ Trimodality therapy has been widely recommended for patients with locally advanced esophageal cancer by most major organizations.^6^, ^7^, ^8^

The prognosis assessment of patients with esophageal cancer receiving neoadjuvant therapy plus esophagectomy is crucial for making postoperative treatment and surveillance strategies, and designing clinical trials. Several studies have showed that histopathologic tumor regression grading is a reliable and reproducible predictor of survival,^9^, ^10^, ^11^ but it only focuses on the primary tumor response. The grading system fails to evaluate the response and status of metastatic lymph nodes after neoadjuvant treatment.

The seventh edition of the American Joint Committee on Cancer (AJCC) staging system for esophageal cancer recommends to classify these patients in accordance with those undergoing esophagectomy alone, and to use y prefix to indicate the cases receiving neoadjuvant therapy. Nevertheless, this system is based on the findings from patients undergoing esophagectomy alone.^12^, ^13^ A major advancement of the eighth edition of the staging system is the introduction of the postneoadjuvant pathologic stage groups, which is a great achievement of international collaboration.^14^ So far, studies validating the postneoadjuvant pathologic staging system have been quite limited; therefore, the prognostic power remains unclear.

Hence, we used a large population from the Surveillance, Epidemiology, and End Results (SEER) database, in order to evaluate whether the novel postneoadjuvant pathologic staging system could distinguish the survival of patients treated with radio(chemo)therapy followed by surgery.

## 2 MATERIALS AND METHODS

### 2.1 Patients

**Figure 1:**
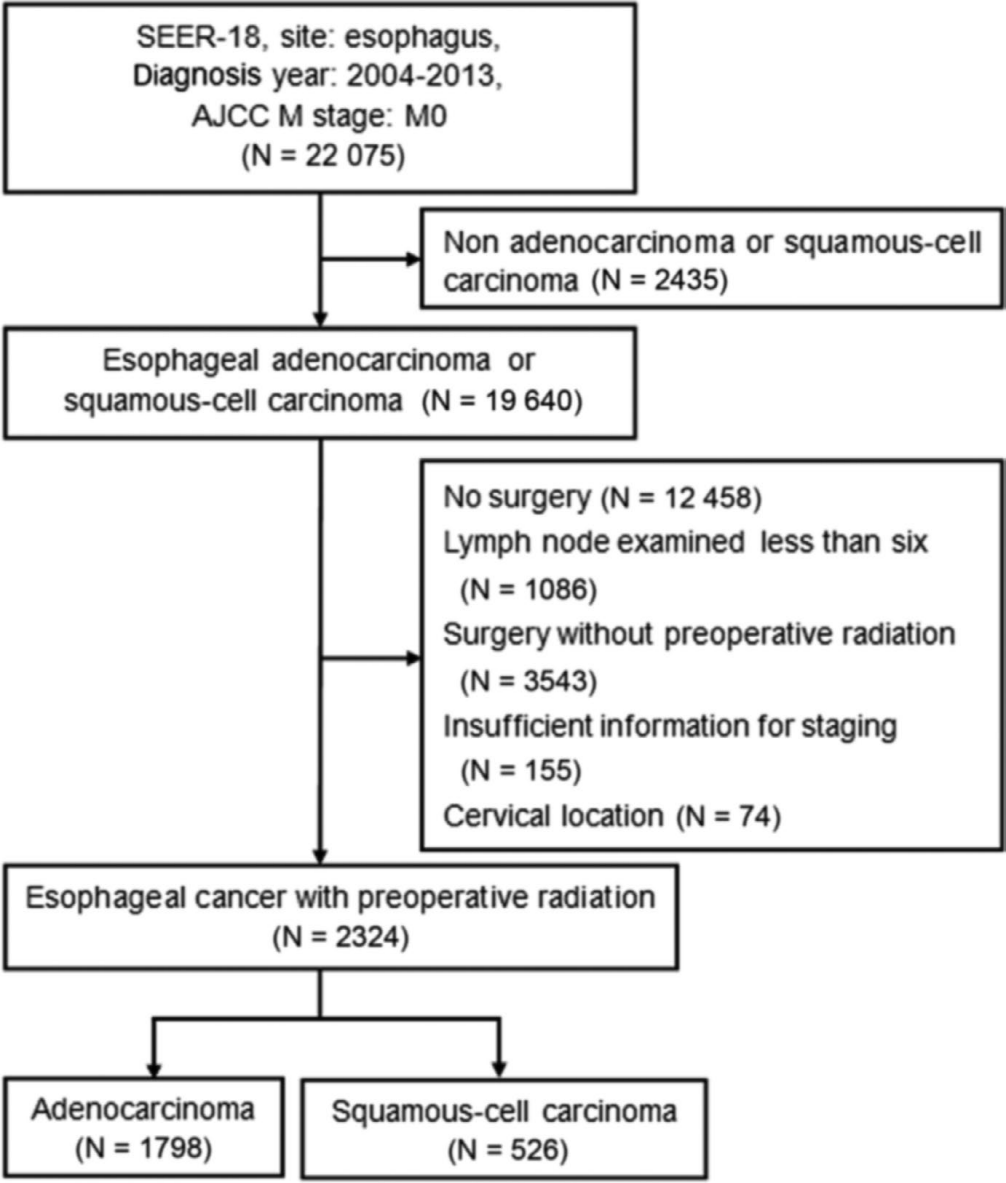
Flow chart of patient selection

The SEER registry is the largest population‐based database of oncology patients in the United States, which covered approximately 28% of the US patients with cancer. We examined the individuals from the SEER 18 Registries Research Data, November 2015 submission (1973‐2013) database, who were diagnosed with nonmetastatic esophageal cancer between 2004 and 2013. Inclusion criteria were: (a) histologically confirmed EAC or ESCC; (b) undergoing surgery of primary site (Code 20‐80); (c) no less than six lymph nodes examined, except T1N0 disease which might undergo local excision; (d) receiving radiation prior to surgery. Exclusion criteria were: (a) insufficient information for staging; (b) cervical location. The flow chart of patient selection was displayed in Figure 1. The Qilu Hospital of Shandong University (Qingdao) Institutional Review Board considered the study exempt.

Patients were restaged according to the eighth edition in the analysis. Those with inadequate available staging information were excluded. Because the majority of patients were white, we designated race as white or others. The recommended number of lymph node examination were 6 in the sixth edition of staging system and 12 in the seventh edition, so we excluded patients whose examined nodes were less than six. Lymph nodes examined were categorized dichotomously as “<12” or “≥12”.

### 2.2 Statistical analysis

We used the SEER*Stat software version 8.3.2 to obtain the SEER database (1973‐2013) through online access. The primary outcomes were overall survival (OS) which was defined as the times (in months) from diagnosis to death due to any cause. The survival times were censored at the time of last follow‐up for live patients or at the time of death from any cause. Median survival time (95% confidence interval [CI]) and survival curves were estimated by Kaplan‐Meier Method. The Cox proportional hazards regression model was applied for estimating hazard ratios (HRs) for OS, and for identifying independent predictor for survival. All the baseline clinicopathologic variables were included in the multivariable Cox regression analysis. Statistical analyses were carried out using SPSS 22.0 for Windows software (SPSS). We used R software (version 3.5.1) to perform time‐dependent ROC analysis (risksetROC package) and compare the *C*‐index (CsChange package), to assess and compare the model discrimination power. Statistical significance was set at 0.05 (two‐sided).

## 3 RESULTS

A total of 22 075 cases with nonmetastatic esophageal cancer were identified from 2004 to 2013. Finally, 2324 cases receiving radio(chemo)therapy prior to surgery were eligible for analysis after selection according to the inclusion and exclusion criteria, including 1798 EACs and 526 ESCCs (Figure 1). The baseline clinical and pathologic characteristics of included patients are summarized in Table 1. There were predominances of males over females, and white race over others. The largest proportion of patients was diagnosed with ypT3 tumors (57.7%), while ypN1 lymph node category was most prevalent (46.5%). According to the eighth edition ypTNM grouping, ypIIIB was the most common stage grouping (41.5%), followed by ypI (23.6%); in the seventh edition TNM grouping, IIIA was the most common one (32.8%), and followed by IIB (28.9%). The median number of lymph nodes examined was 14 (interquartile range, 9‐21).

**Table 1:** Clinical and pathologic characteristics of patients with esophageal cancer receiving preoperative therapy

Characteristics	Total		Adenocarcinoma		Squamous‐cell carcinoma	
	N = 2324	%	N = 1798	%	N = 526	%
Age ≥ 65 y	949	40.8	746	41.5	203	38.6
Sex (Male)	1967	84.6	1631	90.7	336	63.9
Race (White)	2126	91.5	1740	96.8	386	73.4
ypT category						
ypT1	481	20.7	343	19.1	138	26.2
ypT2	383	16.5	294	16.4	89	16.9
ypT3	1341	57.7	1069	59.5	272	51.7
ypT4	119	5.1	92	5.1	27	5.1
ypN category						
ypN0	897	38.6	660	36.7	237	45.1
ypN1	1080	46.5	818	45.5	262	49.8
ypN2	272	11.7	248	13.8	24	4.6
ypN3	75	3.2	72	4.0	3	0.6
Tumor grade						
G1/X	390	16.8	289	16.0	101	19.2
G2/G3	1934	83.4	1509	84.0	425	80.8
Tumor location						
Upper/Middle	332	14.3	79	4.4	253	48.1
Lower	1829	78.7	1614	89.8	215	40.9
Unknown	163	7.0	105	5.8	58	11.0
ypTNM grouping (eighth edition)						
ypI	549	23.6	353	19.6	196	37.3
ypII	333	14.3	295	16.4	38	7.2
ypIIIA	306	13.2	232	12.9	74	14.1
ypIIIB	964	41.5	773	43.0	191	36.3
ypIVA	172	7.4	145	8.1	27	5.1
TNM grouping (seventh edition)						
IA	173	7.4	144	8.0	29	5.5
IB	240	10.3	154	8.6	86	16.3
IIA	104	4.5	52	2.9	52	9.9
IIB	671	28.9	530	29.5	141	26.8
IIIA	762	32.8	590	32.8	172	32.7
IIIB	200	8.6	183	10.2	17	3.2
IIIC	174	7.5	145	8.1	29	5.5
Lympn nodes examined ≥ 12	1381	59.4	1090	60.6	291	55.3

### 3.1 Comparison of the seventh and eighth staging system

**Figure 2:**
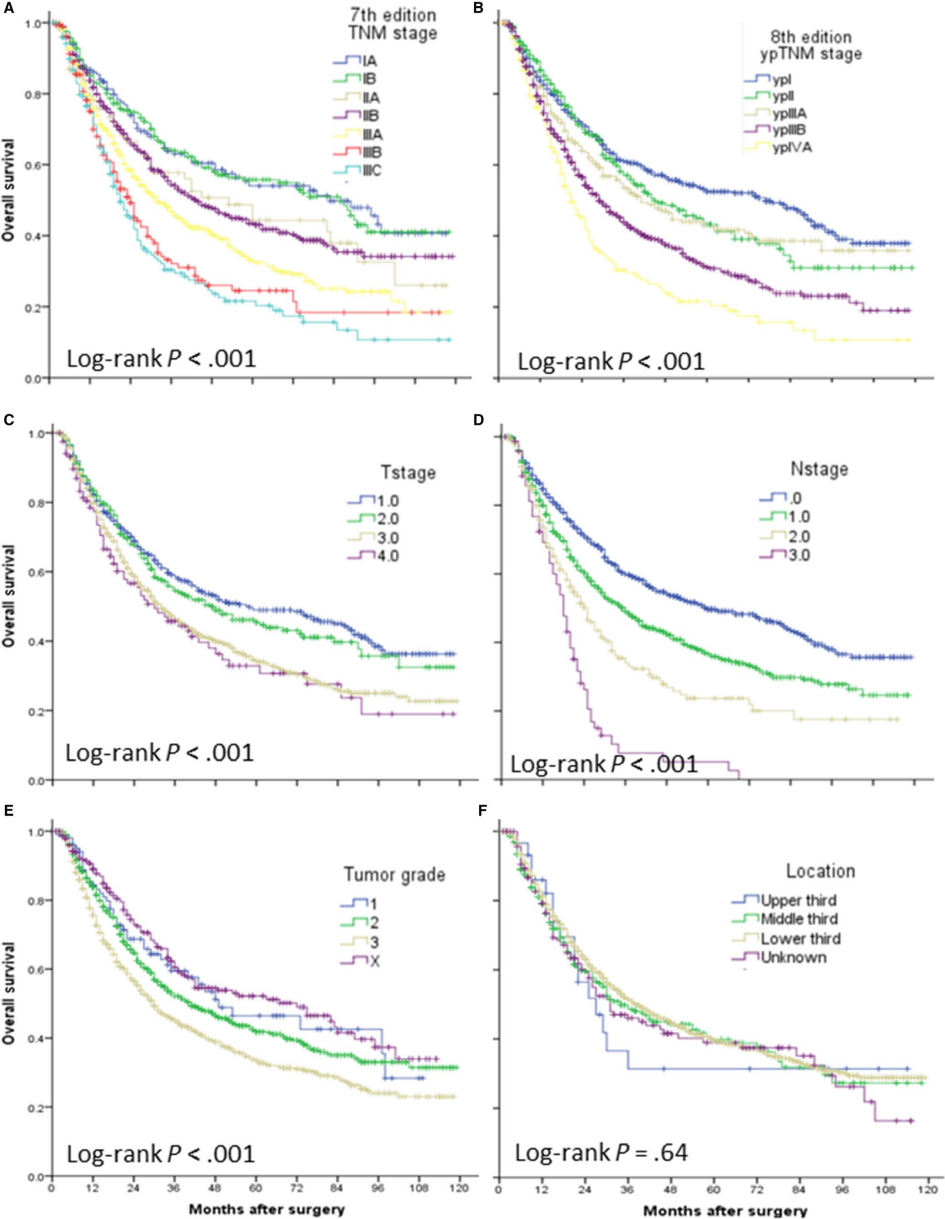
Overall survival of patients with esophageal cancer receiving preoperative radio(chemo)therapy stratified by the seventh edition TNM stage(A), eighth edition ypTNM stage (B), T category (C), N category (D), tumor grade (E), and tumor location (F)

**Figure 3:**
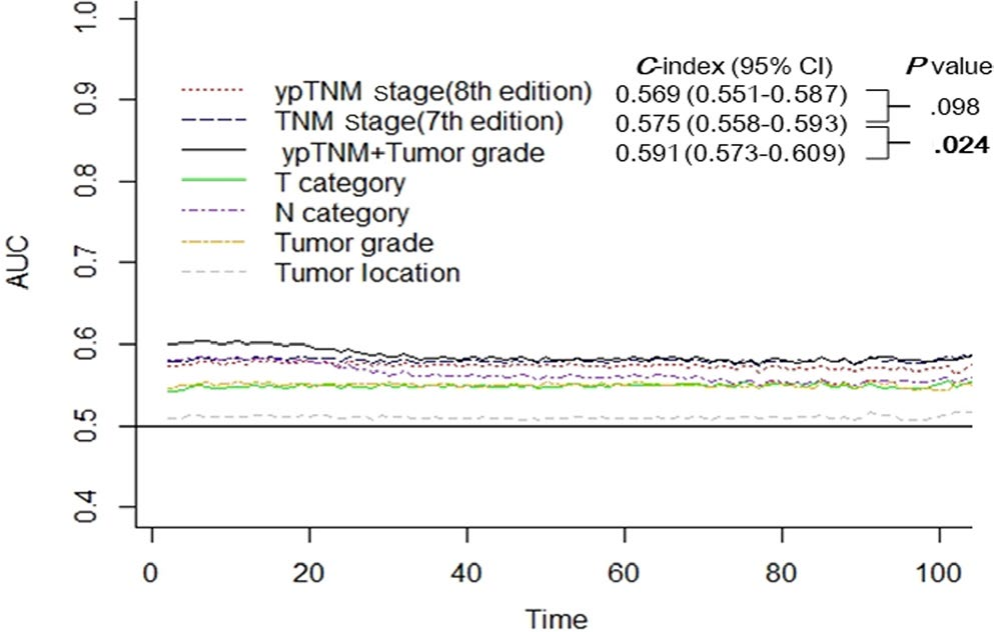
The performance of the seventh edition TNM stage, eighth edition ypTNM stage, ypTNM + tumor grade, and the T, N, G, L categories were compared with time‐dependent receiver operating characteristic (ROC) curves and Harrell concordance index (C‐index). *P* values of the C‐indexe comparison were calculated with the Z‐test

In the seventh edition TNM grouping (Figure 2A) and the eighth edition ypTNM grouping (Figure 2B), statistically significant differences were found in survival curve (both log‐rank *P* < .001). It was noted that, however, the curves of stage IA and IB, IIA and IIB, IIIB, and IIIC were quite close in the seventh edition TNM grouping. Although the eighth edition ypTNM staging simplified as stage I, II, IIIA, IIB, and IVA, overlapping existed in stage I and II at the first 36 months and in stage II and IIIA at about 60‐72 months. The time‐dependent ROC analysis showed that the area under the curve (AUC) of these two staging systems were close during the follow‐up of patients receiving preoperative therapy followed by surgery (Figure 3). We calculated the *C*‐index to quantify the prognostic power of the seventh edition TNM grouping (0.575; 95%CI, 0.558‐0.593) and the eighth edition ypTNM grouping (0.569; 95%CI, 0.551‐0.587), and the difference did not reach statistical significance (*P* = .098).

### 3.2 Survival analysis

We further investigated the prognostic value of T, N, grade, and location categories, respectively. Overall survival significantly decreased with increasing ypT (Figure 2C), ypN (Figure 2D) and histologic grade categories (Figure 2E) (all log‐rank *P* < .001), while tumor location failed to stratify the survival (Figure 2F, log‐rank *P* = .64). The survival plots showed overlapping curves between ypT1 and ypT2, between ypT3 and ypT4, and between G1 and Gx; while ypN category showed a relatively ordered monotone distribution of survival. In the multivariable Cox regression model, including age, gender, race, and number of lymph node examined (Table 2), ypN category was the strongest predictor of overall survival (*P* < .001). Higher tumor grade was also significantly associated with unfavorable outcome (HR, 1.33; 95%CI, 1.12‐1.56; *P* = .001). Overall, ypT category was an independent predictor of survival (*P* = .16), but ypT2 was not significantly associated with survival compared with ypT1. This supported combination of ypT1 and ypT2 in the eighth edition ypTNM grouping to simplify the staging system. These findings were further validated in the EAC (Figure S1) and ESCC (Figure S2) subgroups, and Table S1 displayed the results of multivariable Cox analysis.

**Table 2:** Multivariable Cox analysis for overall survival among esophageal cancer patients after preoperative therapy

Variables	HR	95%CI	*P*
ypT category			.16
ypT1	1.00	—	—
ypT2	1.06	0.86‐1.30	.59
ypT3	1.27	1.08‐1.50	.004
ypT4	1.28	0.96‐1.72	.091
ypN category			<.001
ypN0	1.00	—	—
ypN1	1.37	1.19‐1.58	<.001
ypN2	1.97	1.61‐2.41	<.001
ypN3	3.67	2.76‐4.89	<.001
Tumor grade (G2/G3 vs G1/X)	1.33	1.12‐1.58	.001
Tumor location			.14
Lower	1.00	—	—
Upper/Middle	1.17	0.97‐1.40	.095
Unknown	1.16	0.93‐1.45	.20
Age (≥65 vs < 65)	1.36	1.20‐1.53	<.001
Sex (Female vs Male)	0.80	0.67‐0.96	.014
Race (Other vs White)	1.30	1.06‐1.61	.014
Lymph nodes examined(≥12 vs < 12)	0.79	0.70‐0.89	<.001

### 3.3 Tumor grade improved the prognostication

As the multivariable Cox analysis demonstrated that tumor grade remained an independent predictor for survival in the setting of neoadjuvant therapy, we hypothesized that incorporating tumor grade could also improve the performance of ypTNM staging. As shown in Figure 3, AUC curve of combining ypT, ypN, and ypG categories remained the highest during the follow‐up. The *C*‐index was 0.591 (95%CI, 0.573‐0.609), and was significantly higher than that of the seventh edition TNM staging (*P* = .024).

## 4 DISCUSSION

The current study analyzed 2324 cases with esophageal cancer receiving preoperative radio(chemo)therapy plus surgery in the SEER database from 2004 to 2013. Although ypT category was demonstrated to an independent predictor for survival, ypT2 was not significantly associated with survival compared with ypT1, supporting the combination of ypT1 and ypT2 in the eighth edition ypTNM grouping to simplify the staging system. Lymph node status (ypN category) was the strongest prognostic factor in the neoadjuvant setting for esophageal cancer. Tumor grade category was also an independent predictor of survival, and the addition of grade category could significantly improve the performance of ypTNM staging system.

The development of the eighth edition of AJCC staging system for esophageal cancer was an international effort of the Worldwide Esophageal Cancer Collaboration (WECC). The staging was based on 7773 patients’ pathologic assessment of surgical specimen after neoadjuvant therapy from 33 international institutions.^15^ The WECC developed the first ypTNM recommendations for cancer of the esophagus and esophagogastric junction for the eighth edition AJCC Cancer Staging Manual. The validation of this ypTNM staging system has been scare so for.^16^ The prognostication of these patients is of great importance, because trimodality therapy has become the standard care for locally advanced disease, and has been widely used all over the world. Accurate prognostication allows personalized postoperative treatment, comparison of novel treatment modalities with the standard ones, and communication among different institutions.^17^, ^18^

The criterion of a staging system is that survival should be distinct and monotonically decrease with increasing stage grouping. Despite the statistical significance in Kaplan‐Meier analysis, unfortunately, survival curves of ypI and ypII almost overlapped at the first 36 months, and curves of ypII and ypIIIA groups crossed at about postoperative month 60‐72. Time‐dependent ROC analysis and *C*‐index comparison showed not significant difference in the performance of seventh edition TNM grouping and eighth edition ypTNM grouping. The major finding of the current study was that tumor grade remained an independently predictor for overall survival among esophageal cancer patients after neoadjuvant. Tumor grade might play an important role in the prognostication of not only in the pathologic staging but also in the postneoadjuvant pathologic staging. The better performance of ypTNM staging including tumor grade category indicated that tumor grade should not be omitted in the postneoadjuvant staging.

Another method to improve the prognostication is to identify other important pathologic feathers. Tumor response to neoadjuvant treatment has been demonstrated to be more predictive of outcomes than the depth of invasion by several studies.^9^, ^19^ Dr Holscher and his colleagues proposed a combined classification of primary tumor remission and lymph node status, which represented a simple and reproducible prognostic classification of the effect of neoadjuvant treatment in EAC.^10^ So far, there has been no agreement on the histologic remission grading, and its prognostic effects remains controversial.^11^, ^19^, ^20^ Additionally, our previous study^21^ showed that lymphovascular invasion (LVI), which was an essential step in dissemination of cancer cells, was associated with increased mortality of patients with esophageal cancer undergoing esophagectomy, in accordance with studies from the West.^22^, ^23^ Furthermore, Dr Chen et al^24^ reported LVI could be easily evaluated in specimen after radiochemotherapy, and was independently associated with shorter survival in these patients, indicating that LVI might provide new clues for the prognostic stratification.

The current study also revealed that ypN category from N0 to N3 could effectively stratify the survival of patients with esophageal cancer after esophageal cancer. Previous studies showed that nodal status was an important prognostic factor for esophageal patients receiving neoadjuvant therapy.^19^, ^24^, ^25^ These studies were habitually based on the sixth edition, and nodal status was simply categorized dichotomously as node negative or positive. Our data highlighted the prognostic significance of the number of positive lymph nodes. Neoadjuvant radiochemotherapy could alter the frequency, localization and pattern of metastatic lymph nodes of esophageal cancer,^26^ and the optimal extent of lymphadenectomy has not been defined yet. Using 12 lymph nodes examined as cutoff point, our study found that more aggressive lymphadenectomy could improve survival after neoadjuvant therapy, in accordance with the findings among patients undergoing surgery alone.^27^, ^28^, ^29^ This suggested that sufficient lymphadenectomy is not only necessary for accurate staging but also beneficial for survival. On the contrary, retrospective analyses of two European randomized trials showed that the number of examined nodes was not predictor of survival after neoadjuvant radiochemotherapy.^30^, ^31^ Controversy about the frequency and distribution of lymph node metastasis requires more investigations.

This study had some noteworthy limitations inherent to the SEER database. First of all, the database contained no information about the usage and regimen of chemotherapy, and only patients receiving radiation prior surgery were eligible. A survey of national practice found that only 0.1% of patients were treated with neoadjuvant radiation alone, and 27% received chemoradiation prior to surgery.^32^ We believe it is safe to take preoperative radiation as a surrogate for nCRT in SEER database. Additionally, the interval between preoperative therapy and surgery was also unavailable. Patients underwent salvage resection for recurrent disease could not been excluded, although they only accounted for a small proportion (<10%).^33^, ^34^, ^35^ Another limitation of this data set is the lack of coding to consistently report response to neoadjuvant therapy, and identify pathologic complete responders, who might be classified as unstaged (Tx, Nx). Lastly, the database lacked of information regarding clinical staging, surgical margins, or disease recurrence.

In conclusion, our analysis based on the national SEER database revealed that lymph node status (ypN category) remained an important prognostic factor, and supported the simplification of the ypTNM grouping by combining ypT1 and ypT2. Tumor grade was an independent predictor of overall survival, and could improve the performance of ypTNM staging. If this was validated in the future study, tumor grade might be considered to be included in the ypTNM staging system.

## AUTHOR CONTRIBUTION

QYH and YGY were involved in conception and design of the study. YGY, MG, and XLH were involved in acquisition, analysis, or interpretation of data. QYH, YGY, and M.G were involved in manuscript writing and revision. All authors were involved in approval of the manuscript.

## Supporting information

Supplementary Material

## Data Availability

All data examined in this study were obtained from the SEER database with the purpose of research. The data are available from the corresponding author upon reasonable request.
