# Supplementary material for: Evaluating the eighth edition TNM staging system for esophageal cancer among patients receiving neoadjuvant therapy: A SEER study
Source: Cancer Med. 2020 May 11;9(13):4648–55. doi: 10.1002/cam4.2997 (PMC7333840; doi:10.1002/cam4.2997)
Supplement: Supplementary file 1 — Supplementary Material [file CAM4-9-4648-s001.docx]

**Supplementary materials**

**Evaluating the 8^th^ edition TNM staging system for esophageal cancer among patients receiving neoadjuvant therapy: a SEER study**


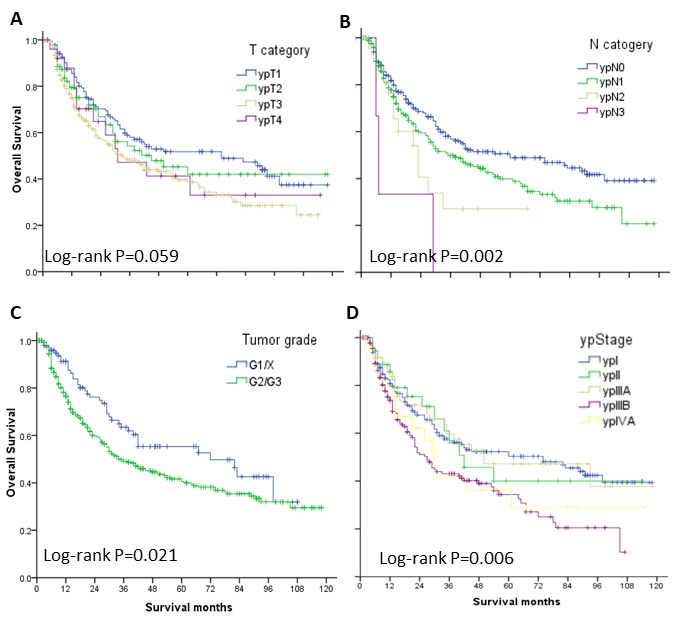


Supplementary Figure S1. Overall survival of patients with esophageal adenocarcinoma receiving preoperative radio(chemo)therapy stratified by ypT category (A), ypN category (B), tumor grade (C) and ypTNM staging groups (D).


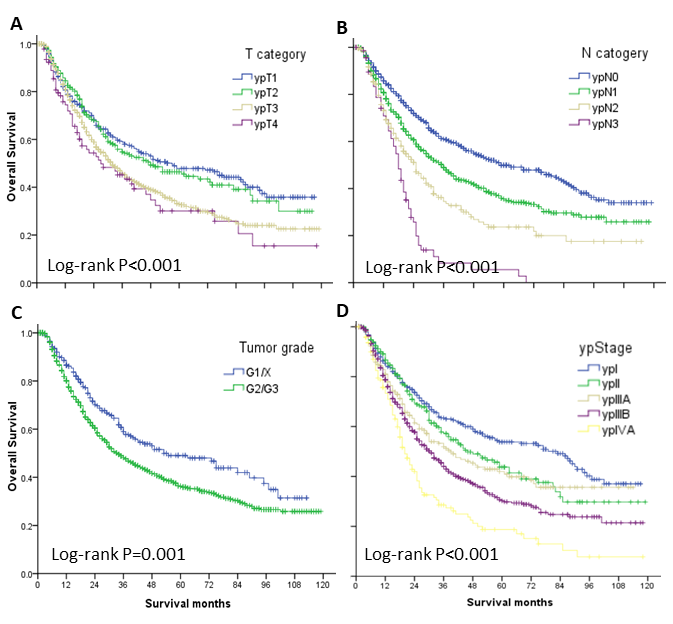


Supplementary Figure S2. Overall survival of patients with esophageal squamous cell cancer receiving preoperative radio(chemo)therapy stratified by ypT category (A), ypN category (B), tumor grade (C) and ypTNM staging groups (D).

Supplementary Table S1. Multivariable analysis for overall survival among esophageal cancer patients after preoperative therapy

| **Variables** | **Adenocarcinoma** | | |  | **Squamous-cell carcinoma** | | |
| --- | --- | --- | --- | --- | --- | --- | --- |
|  | **HR** | **95%CI** | ***P*** |  | **HR** | **95%CI** | ***P*** |
| **ypT category** |  |  | 0.42 |  |  |  | 0.19 |
| **ypT1** | 1.00 | - | - |  | 1.00 | - | - |
| **ypT2** | 1.00 | 0.79-1.27 | 0.98 |  | 1.26 | 0.82-1.92 | 0.29 |
| **ypT3** | 1.23 | 1.02-1.50 | 0.033 |  | 1.43 | 1.02-1.99 | 0.036 |
| **ypT4** | 1.35 | 0.97-1.88 | 0.072 |  | 1.09 | 0.58-2.05 | 1.09 |
| **ypN category** |  |  | <0.001 |  |  |  | 0.014 |
| **ypN0** | 1.00 | - | - |  | 1.00 | - | - |
| **ypN1** | 1.38 | 1.18-1.63 | <0.001 |  | 1.31 | 0.98-1.74 | 0.067 |
| **ypN2** | 1.98 | 1.59-2.46 | <0.001 |  | 1.85 | 1.01-3.38 | 0.045 |
| **ypN3** | 3.67 | 2.72-4.96 | <0.001 |  | 4.51 | 1.40-14.5 | 0.011 |
| **Tumor grade**  **(G2/G3 vs. G1/X)** | 1.31 | 1.08-1.60 | 0.007 |  | 1.36 | 0.96-1.92 | 0.086 |
| **Tumor location** |  |  | 0.35 |  |  |  | 0.20 |
| **Lower** | 1.00 | - | - |  | 1.00 | - | - |
| **Upper/Middle** | 1.25 | 0.90-1.72 | 0.19 |  | 1.17 | 0.89-1.54 | 0.25 |
| **Unknown** | 1.10 | 0.84-1.45 | 0.50 |  | 1.43 | 0.95-2.16 | 0.089 |
| **Age (≥65 vs. <65)** | 1.41 | 1.23-1.61 | <0.001 |  | 1.21 | 0.93-1.58 | 0.15 |
| **Sex (Female vs. Male)** | 0.80 | 0.63-1.02 | 0.077 |  | 0.83 | 0.62-1.10 | 0.18 |
| **Race (Other vs. White)** | 1.09 | 0.74-1.62 | 0.66 |  | 1.44 | 1.09-1.91 | 0.011 |
| **Lymph nodes examined(≥12 vs. <12)** | 0.80 | 0.70-0.92 | 0.002 |  | 0.74 | 0.57-0.97 | 0.026 |
